# Integrating BERT pre-training with graph common neighbours for predicting ceRNA interactions

**DOI:** 10.3389/fgene.2025.1606016

**Published:** 2025-09-03

**Authors:** Zhengxing Xie, Tianping Ying, Ge Jing, Shiyang Liang, Junhua Liu, Lianghua Tang

**Affiliations:** ^1^ Guizhou University of Traditional Chinese Medicine, Guiyang, Guizhou, China; ^2^ The Second Affiliated Hospital of Guizhou University of Traditional Chinese Medicine, Guiyang, Guizhou, China; ^3^ Department of Internal Medicine, The No. 944 Hospital of Logistic Support Force of PLA, Jiuquan, Gansu, China; ^4^ School of Computing and Information Systems, The University of Melbourne, Melbourne, VC, Australia

**Keywords:** lncRNA, circRNA, miRNA, ceRNA, pre-train, graph neural network

## Abstract

**Introduction:**

Predicting interactions between microRNAs (miRNAs) and competing endogenous RNAs (ceRNAs), including long non-coding RNAs (lncRNAs) and circular RNAs (circRNAs), is essential for understanding gene regulation. With the development of Graph Neural Networks (GNNs), existing works have demonstrated the ability to capture information from miRNA-ceRNA interactions to predict unseen associations. However, current deep GNNs only leverage node-node pairwise features, neglecting the information inherent in the RNA chains themselves, as different RNAs possess chains of varying lengths.

**Methods:**

To address this issue, we propose a novel model termed the BERT-based ceRNA Graph Predictor (BCGP), which leverages both RNA sequence information and the heterogeneous relationships among lncRNAs, circRNAs, and miRNAs. Our BCGP method employs a transformer-based model to generate contextualized representations that consider the global context of the entire RNA sequence. Subsequently, we enrich the RNA interaction graph using these contextualized representations. Furthermore, to improve the performance of association prediction, BCGP utilizes the Neural Common Neighbour (NCN) technique to capture more refined node features, leading to more informative and flexible representations.

**Results:**

Through comprehensive experiments on two real-world datasets of lncRNA-miRNA and circRNA-miRNA associations, we demonstrate that BCGP outperforms competitive baselines across various evaluation metrics and achieves higher accuracy in association predictions. In our case studies on two types of miRNAs, we show BCGP’s remarkable performance in predicting both miRNA-lncRNA and miRNA-circRNA associations.

**Discussion:**

Our findings demonstrate that by integrating RNA sequence information with interaction relationships within the graph, the BCGP model significantly enhances the accuracy of association prediction. This provides a new computational tool for understanding complex gene regulatory networks.

## 1 Introduction

MicroRNAs (miRNAs) are a class of small, non-coding RNA molecules that play a crucial role in the regulation of gene expression (Ye et al., 2019). MiRNAs are present in plants, animals and some viruses and they can significantly affect a broad range of biological processes (Bartel, 2018). Specifically, they primarily regulate gene expression through binding to the 3′ untranslated regions (*UTRs*) of target mRNAs, leading to their degradation or inhibition of translation. The degree of complementarity between the miRNA and the target mRNA is crucial, as it determines the mechanism of repression. MiRNA sponges, also known as competing endogenous RNAs (ceRNAs), embody a sophisticated biological mechanism that serves to regulate the activity of miRNAs within cellular environments (Alkan and Akgül, 2022). Such a mechanism leverages RNA molecules containing multiple miRNA binding sites to effectively “absorb” or “sponge” certain miRNAs. As a result, it diminishes the suppressive impacts of these miRNAs on their intended target mRNAs. There are numerous previous studies that have employed machine learning to forecast the miRNA-disease associations, achieving satisfactory results (Chen et al., 2021; Ha et al., 2020).

Long non-coding RNAs (lncRNAs), a unique type of RNA with over 200 nucleotides, do not have protein-coding capacity. Circular RNAs (circRNAs) constitute another category of non-coding RNA, distinguished by their unique covalently closed-loop configuration. Unlike linear RNAs, circRNAs lack both a 5′ cap and a 3′ poly-A tail in their sequences. They are synthesized through a mechanism known as back-splicing, wherein a splice donor site downstream is connected to a splice acceptor site upstream, resulting in the circularization of the RNA molecule (Kristensen et al., 2022). Both lncRNA and circRNA are considered as the major types of ceRNAs, where they can sponge specific miRNAs when they have miRNA binding sites, which can potentially alleviate the inhibitory effects these miRNAs exert on their target mRNAs.

Inspired by methods for predicting miRNA-disease associations (Ha et al., 2019), several approaches for ceRNA association prediction have emerged in recent years. For example, Zhu et al. (2017) discovered that lnc-mg promotes myogenesis by sponging microRNA-125b to regulate the abundance of the IGF2 protein. Furthermore, Zhang et al. (2018) identified that lncRNA MAR1 acts as a miR-487b sponge to promote skeletal muscle differentiation and regeneration. Similarly, Yang et al. (2018) found that circ-ITCH can sponge miR-17 and miR-224, thereby upregulating the expression of p21 and PTEN, which in turn suppresses the aggressive biological behaviors associated with bladder cancer.

Traditionally, determining the associations between miRNA and ceRNAs requires the use of technologies such as High-Throughput Sequencing (Li et al., 2018), RNA Immunoprecipitation (Gawronski et al., 2018) and Dual-Luciferase Reporter (Cao et al., 2016). However, these methods can cost a large investment of time and resources. With the advancement of deep learning technologies especially the deep Graph Neural Networks (GNNs) (Kipf and Welling, 2016) and the accumulation of historical experimental data, there has been a surge in efforts to predict lncRNA-miRNA and circRNA-miRNA associations using computational techniques. For example, Wang W. et al. (2022) proposed a model, named GCNCRF, to predict lncRNA-miRNA associations based on the graph convolutional network (GCN) and conditional random field. Furthermore, a recent work (Wang Z. et al., 2023) has employed a sequence pre-training-based Graph Neural Network to predict associations between lncRNAs and miRNAs.

Although effective, existing methods still have several limitations. First, most of the graph-based models have not effectively utilized the information contained within RNA sequences. As the foundational elements, an RNA sequence contains nearly all the information of the RNA (Charles Richard and Eichhorn, 2018). Using an appropriate sequential model to analyze these RNA sequences could uncover enormous characteristics of each RNA. Furthermore, most previous studies used only homogeneous or bipartite graphs to predict the associations between lncRNA-miRNA or circRNA-miRNA. However, according to the mechanism of ceRNAs (Zhong et al., 2018), circRNAs and lncRNAs, although two different types of RNAs, both function as miRNA sponges within this network. Therefore, a heterogeneous graph can be used to construct the ceRNA network among circRNA, lncRNA, and miRNA to further boost the performance.

To address the aforementioned challenges, we propose a novel framework, the BERT-based ceRNA Graph Predictor (BCGP), which uniquely integrates sequence-level and structural information for comprehensive ceRNA interaction prediction. Unlike existing methods, BCGP innovatively combines Bidirectional Encoder Representations from Transformers (BERT) for sequence pre-training and a heterogeneous graph model for fine-tuning. Specifically, our framework incorporates heterogeneous relations between lncRNAs, miRNAs, and circRNAs to capture both contextual and relational dependencies. In the pre-training stage, BCGP uses BERT with Masked Language Modeling (MLM) as the training objective, a choice justified by extensive comparative analysis, to derive high-quality embeddings for different types of RNA sequences. These embeddings are then seamlessly integrated into a heterogeneous graph, where nodes represent RNAs and edges capture their intricate relationships. In the fine-tuning stage, BCGP leverages the Neural Common Neighbour (NCN) method (Wang X. et al., 2023), further enhancing the expressive power of the graph embeddings by incorporating relational patterns. Extensive experiments have demonstrated the effectiveness of our novel BCGP framework, consistently outperforming other state-of-the-art models. Our ablation studies confirm the critical contributions of each component within BCGP, while case studies on two specific miRNAs, hsa-miR-143 and hsa-miR-6808-5p, validate the superior practicality and real-world relevance of our approach. By bridging the gap between sequence-level and structural modeling, our work establishes a new paradigm for ceRNA interaction prediction.

## 2 Materials and methods

### 2.1 Overview

**FIGURE 1:**
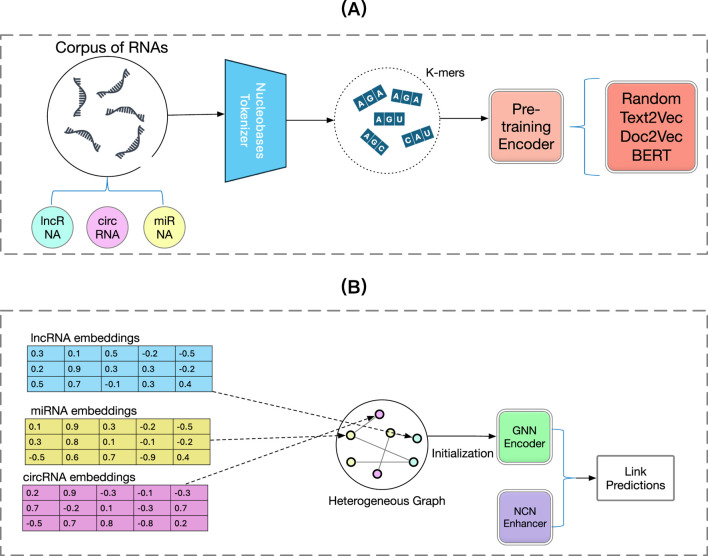
Overview framework of the proposed BCGP model. In the pre-training stage, BCGP first tokenizes an RNA sequence with k -mers and utilizes the BERT model to learn contextualized embeddings for each RNA sequence. In the fine-tuning stage, BCGP leverages GNN and Neural Common Neighbour (NCN) to capture the complex relationship and learn informative node representations. **(A)** Pre-training stage. **(B)** Fine-tunning stage.

In this section, we will describe our proposed method BCGP that we use to generate pre-trained embeddings for lncRNAs, circRNAs, or miRNAs and fine-tune with general Graph Neural Networks to recover the unseen associations between them. Additionally, we integrate a novel training method, Neural Common Neighbour (NCN) (Wang X. et al., 2023) to further enhance the performance of the fine-tuning GNNs. In Section 2.2, we will present the notations used in this article and briefly describe our research problem formulation. Second, in Section 2.3, we demonstrate the pre-training stage of BCGP, where we use the k -mers method to split all RNA sequences into fragments of equal length, then we use Bidirectional Encoder Representations from Transformers (BERT) (Devlin et al., 2018), which is a transformer-based contextualized language representation model to generate pre-trained embeddings. Third, in Section 2.4, we describe a general fine-tuning method to incorporate a GNN to leverage the pre-trained embeddings obtained from Section 2.3. To boost the performance of the fine-tuning GNN, we also present the integration of a novel training method named Neural Common Neighbour. We use Figure 1 to illustrate the overview of our BCGP method. The detailed mathematical formulation of our model is provided in Equations 1–13.

### 2.2 Preliminaries

To initialize our task, we first define all entities and their associated information. In particular, we denote sets of lncRNAs, circRNAs and miRNAs as $V_{\text{lnc}}$ , $V_{\text{circ}}$ and $V_{\text{mi}}$ respectively. Considering the associations between lncRNAs, circRNAs, and miRNAs we construct an undirected graph G={V,E,A} , where V is the set of all RNAs $\left(V=V_{\text{lnc}}\cup V_{\text{circ}}\cup V_{\text{mi}}\right)$ , E⊆V×V represents the set of associations between RNAs and adjacency matrix $A\in R^{N\times N}$ is a symmetric matrix, which is defined as follows:

$$A_{uv}=\left\{\begin{array}{cc} 1\; & \text{if }\left(u,v\right)\in E,\\ 0\; & \text{otherwise}, \end{array}\right.$$ (1)

where N=|V| is the number of all types of RNAs. The degree of node u is $d\left(u,A\right)≔\sum_{v=1}^{N}A_{uv}$ . The neighbours of node u are the nodes connected to u , which is defined as $Ne\left(u,A\right)≔\left\{v|v\in V,A_{uv}>0\right\}$ . For brevity, we use Ne(u) to denote Ne(u,A) since A is fixed. The common neighbour refers to the nodes connected to i and j : Ne(i)∩Ne(j) . Let S be the collection of RNA sequences, including all lncRNAs, circRNAs and miRNAs. Specifically, $S=\left\{s_{1},s_{2},\ldots ,s_{N}\right\}$ , where each $s_{i}$ represents a unique RNA sequence.

In this next section, we will provide a detailed description of our proposed method, including the techniques in the pre-training and fine-tuning stages.

### 2.3 Pre-training stage

During the pre-training stage, our goal is to effectively project the information of RNAs from their nucleotide sequences to latent embeddings. Specifically, we aim to simplify computational demands while enriching the semantic content of the embeddings, which captures the intrinsic patterns and relations within RNA sequences. Such embeddings not only preserve the biological significance and genetic information of RNA sequences but also serve as the initial features of nodes for the subsequent fine-tuning stage so that those sequence information can be encoded into the graph representation.

Instead of treating each base (A, C, G, U) as an individual token, given an RNA sequence $s_{i}$ , we leverage the k -mers tokenization to segment $s_{i}$ into overlapping and equal-length segment, with k indicating the length of each segment. Let $C=\left\{C_{1},C_{2},\ldots ,C_{N}\right\}$ be the collection of RNA k -mer sequences, and $C_{i}$ can be denoted as follows:

$$C_{i}=\left\{s_{i,\left[j:j+k\right]}|j=1,2,\ldots ,M\right\},$$ (2)

where $M=L_{i}-k+1$ represents the total number of k -mers of $s_{i}$ and $L_{i}$ represents the length of $s_{i}$ . For example, the RNA sequence ‘UAACAC’ can be tokenized to a sequence of four 3-mers: UAA, AAC, ACA, CAC. This method not only enables the implementation of sequence embedding algorithms, but also deepens the understanding of richer contextual information for each nucleic acid sequence.

After tokenizing each RNA sequence into the overlapping segment using k -mer tokenization, we utilize BERT to capture both contextual and structural information from the whole corpus of RNA sequences with an attention mechanism. As a widely used transformer-based language representation model, BERT enables the generation of contextualized representations that consider the global context of the entire sequence, allowing the identification of the intricate patterns and relationships within the RNA sequences.

Given a sequence of k -mers $C_{i}$ derived from an RNA sequence $s_{i}$ , we first initialize each k -mer into a high-dimensional vector through an embedding process, resulting in the embedding matrix $X_{i}\in R^{M\times D}$ , where D denotes the dimension of the embedding vectors. To obtain contextual and informative embedding $Z_{i}$ , BCGP performs the multi-head self-attention mechanism on $X_{i}$ , which is defined as:

$$\text{MultiHead}\left(X_{i}\right)=⊕\left({\text{head}}_{i1},{\text{head}}_{i2},\ldots ,{\text{head}}_{iH}\right)W^{O},$$ (3)

$${\text{head}}_{ih}=\text{softmax}\left(\frac{Q_{ih}K_{ih}^{T}}{\sqrt{d_{k}}}\right)V_{ih},$$ (4)

$$\text{where}\;\;Q_{ih}=X_{i}W_{h}^{Q},\;\;K_{ih}=X_{i}W_{h}^{K},\;V_{ih}=X_{i}W_{h}^{V},$$ (5)

where ⊕ denotes the concatenation operation, $W^{O}$ , $W_{h}^{Q}$ , $W_{h}^{K}$ and $W_{h}^{V}$ are the learnable parameters for linear projection for the h -th head. Here, H represents the number of attention heads, and $d_{k}$ is the scaling factor used to maintain numerical stability and facilitate stable gradients during training, often set to D/H . After applying the multi-head self-attention mechanism multiple times, we can derive the contextual embedding $Z_{i}$ from the output of the last layer, which is denoted as:

$$Z_{i}={\text{MultiHead}}_{\text{Last}}\left(X_{i}\right).$$ (6)

Building upon the aforementioned self-attention mechanism, we adapt the Masked Language Modeling (MLM) to train the BERT model on RNA sequences. For each RNA sequence, we randomly select regions constituting 15% of the sequence and mask contiguous k -mers within these regions. Using the surrounding context, the model is then trained to predict the masked k -mers. The training objective to minimize the cross-entropy loss which is defined as follows:

$$L_{\text{MLM}}=-\overset{T}{\underset{i=1}{\sum }}y_{i}^{\prime }\log\left(y_{i}\right),$$ (7)

where $y_{i}^{\prime }$ represents the one-hot encoded ground-truth vector for the masked k -mers and $y_{i}$ denotes the predicted probability distribution over the k -mer vocabulary for each of the T masked positions. Specifically, the predicted probability distribution $y_{i}$ for a masked position is defined as follows:

$$y_{i}=\text{Softmax}\left(z_{i}W_{c}+b_{c}\right),$$ (8)

where $z_{i}$ is the i -th encoded representation from $Z_{i}$ , $W_{c}$ and $b_{c}$ are the parameters of the linear classifier respectively.

### 2.4 Fine-tuning stage

After obtaining the initial RNA embeddings for lncRNAs, circRNAs and miRNAs from BERT during the pre-training stage, we construct a heterogeneous graph. This graph integrates known associations between lncRNAs and miRNAs, as well as the high-quality associations between circRNAs and miRNAs, with these relationships represented as edges. The pre-trained RNA sequence embeddings are utilized as node features in this graph. To obtain effective node embeddings, we leverage GNNs to capture the intricate and complex relationships between entities.

The current *de facto* design of GNNs follows the message passing framework (Kipf and Welling, 2016), which is based on the core idea of recursive neighborhood aggregation. Specifically, for an L -layer GNN, the representation learning function of the l -th layer is represented as:

$$b_{i}^{\left(l\right)}=AGGREGATE\left(\left\{h_{i}^{\left(l-1\right)}\right\}\cup \left\{h_{j}^{\left(l-1\right)}:j\in Ne\left(i\right)\right\}\right),$$ (9)

$$h_{i}^{\left(l\right)}=COMBINE\left(h_{i}^{\left(l-1\right)},b_{i}^{\left(l\right)}\right),$$ (10)

where $b_{i}^{\left(l\right)}$ is a message vector computed from the representations of the neighbors Ne(i) from the previous layer i.e., l−1 -th layer, Ne(i) is a set of nodes adjacent to $v_{i}$ , $h_{i}^{\left(l\right)}$ is the representation of node $v_{i}$ at the l -th layer with $h_{i}^{\left(0\right)}=z_{i}$ , and AGGREGATE(⋅) and COMBINE(⋅) are the component functions of GNN layers. It is worth noting that our proposed BCGP approach is a general framework that can be incorporated with various GNNs.

To further boost the performance of association prediction, we leverage the Neural Common Neighbour (Wang X. et al., 2023) to capture more refined node features such as multi-hop structure and attribute information. Specifically, after obtaining the representation $h_{i}$ of node $v_{i}$ , instead of directly using the node representation for link prediction, BCGP focuses on the pairwise relationships between nodes, specifically leveraging the common neighbours of each pair nodes under consideration. For a target link between node i and node j , BCGP sums up the representation of their common neighbours obtained from the GNNs. This emphasizes the structural context and the shared neighbourhood, which are pivotal for predicting the existence of a link. Formally, the pairwise representation $e_{ij}$ for a potential link between node i and node j can be represented as:

$$e_{ij}=\underset{u\in Ne\left(i\right)\cap Ne\left(j\right)}{\sum }{\text{GNN}}_{\theta }\left(u,A,Z\right),$$ (11)

where θ represents the parameters of the GNN, Ne(i)∩Ne(j) denotes the set of common neighbours between node i and node j . The aggregated pairwise representation $e_{ij}$ is then used to compute the probability ${\hat{y}}_{ij}$ of a link between node i and node j . This is achieved by passing $e_{ij}$ through a final prediction layer, such as a fully connected layer with a sigmoid activation function, which is denoted as follows:

$${\hat{y}}_{ij}=\sigma \left(W_{n}e_{ij}+b_{n}\right),$$ (12)

where σ denotes the sigmoid function, and $W_{n}$ and $b_{n}$ are learnable parameters of the prediction layer.

To train the model on the link prediction (LP) task, we use the binary cross-entropy loss, which is denoted as:

$$L_{\text{LP}}=-\frac{1}{\left|M\right|}\underset{\left(i,j\right)\in M}{\sum }\left[y_{ij}\log\left({\hat{y}}_{ij}\right)+\left(1-y_{ij}\right)\log\left(1-{\hat{y}}_{ij}\right)\right],$$ (13)

where M represents the set of all node pairs, which contains both positive (existing links) and negative (non-existing links) samples, |M| denotes the total number of node pairs in the set M , $y_{ij}$ is the ground truth label for the link between nodes i and j , where $y_{ij}=1$ if a link exists and $y_{ij}=0$ otherwise.

## 3 Results

In this section, we present the evaluation performance of our BCGP. We begin by describing the datasets used in our experiments. Following this, we introduce the experimental results, including the evaluation of pre-training, fine-tuning, performance comparisons with baselines, hyperparameter analysis, and case studies. For more details on the evaluation metrics and experimental settings, please refer to Sections 1, 2 of the Supplementary Material.

### 3.1 Datasets

In this study, we focus on leveraging a comprehensive dataset to model lncRNA-miRNA and circRNA-miRNA associations. We have constructed a total of four datasets: lncRNA-miRNA association 1 (LMA1), circRNA-miRNA associations 1 (CMA1), lncRNA-miRNA association 2 (LMA2), and circRNA-miRNA associations 2 (CMA2). For the lncRNA-miRNA associations in LMA1, based on previous research (Wang Z. et al., 2023), we utilized the LncACTdb 3.0 (Wang P. et al., 2022) database. From this database, we extracted 1,057 experimentally verified lncRNA-miRNA associations, containing 284 lncRNAs and 520 miRNAs. Regarding the circRNA-miRNA associations in CMA1, building upon prior studies (Guo et al., 2024), we used CircBank (Liu et al., 2019) as the primary data source, obtaining 20,771 high-quality associations involving 3,802 circRNAs and 1,273 miRNAs. The lncRNA-miRNA associations in LMA2 were sourced from lncRNASNP V3.0 (Yang et al., 2023), comprising a total of 8,502 lncRNA-miRNA associations, including 467 lncRNAs and 254 miRNAs. As for the circRNA-miRNA associations in CMA2, they were derived from the dataset 1 of the KGANCDA (Lan et al., 2022), with a total of 702 circRNA-miRNA associations, encompassing 471 circRNAs and 439 miRNAs. The sequences of lncRNAs were sourced from LNCipedia (Volders et al., 2019) and NONCODE (Zhao et al., 2021), the sequences of circRNAs were obtained from CircBase (Glažar et al., 2014), and the sequences of miRNAs were acquired from miRBase (Griffiths-Jones et al., 2007).

### 3.2 Examination of pre-training

To rigorously evaluate the performance of pre-training within our method, we compare several pre-training methods commonly used for initializing node embedding in GNN. We include two baselines, namely, the Random Embedding and the Adjacency Matrix Embedding methods which do not consider any RNA sequence information. The Random Embedding method initializes node embeddings with random values generated from a Gaussian distribution, while the Adjacency Matrix Embedding method leverages the adjacency matrix, representing the relationships between nodes in a lncRNA-miRNA-circRNA association graph, to generate node embeddings. Furthermore, we also compare three sequence-specific pre-training methods, including Text2vec (Mikolov et al., 2013), Doc2vec (Le and Mikolov, 2014) and HyenaDNA (Nguyen et al., 2024). Firstly, Text2vec employs a “bag of words” model for the k -mer representations of RNA sequence, converting each lncRNA, circRNA or miRNA sequence into a numerical representation based on k -mer occurrence frequency. Meanwhile, Doc2vec extends the Word2vec algorithm that generates document-level embeddings of the RNA sequences. Additionally, HyenaDNA leverages pre-trained HyenaDNA to generate the embeddings of RNA sequences at the nucleotide level, capturing the long-range dependencies within the RNA sequences.

The experimental results on the LMA1 and CMA1 datasets presented in Table 1, demonstrate the effectiveness of BCGP-BERT. For lncRNA-miRNA prediction, BCGP-BERT achieves the highest scores across all evaluation metrics, with an F1 score of 0.439, AUC of 0.904, AP of 0.435, and NDCG of 0.816, outperforming all other pre-training methods. Similarly, for circRNA-miRNA prediction, BCGP-BERT excels in all metrics, with an F1 score of 0.572, AUC of 0.948, AP of 0.712, and NDCG of 0.957. These results suggest that BCGP-BERT enhances association prediction by learning contextualized representations that capture the global context of entire sequences. Furthermore, the findings also highlight the effectiveness of using the BERT model trained on task-related data through Masked Language Modeling, enabling BCGP-BERT to discover the intricate pattern of RNA sequences and demonstrate its robustness in predicting RNA interactions. Additional pre-training results on the LMA2 and CMA2 datasets are provided in Supplementary Appendix S3.

**TABLE 1:** Overall performance comparison of different pre-training methods fine-tuned with GCN on lncRNA-miRNA and circRNA-miRNA association prediction tasks using the LMA1 and CMA1 datasets.

Methods	lncRNA-miRNA				circRNA-miRNA			
Pre-train	F1	AUC	AP	NDCG	F1	AUC	AP	NDCG
BCGP-Random	0.397	0.891	0.397	0.801	0.500	0.921	0.591	0.933
BCGP-Text2vec	0.428	0.900	0.425	0.809	0.550	0.928	0.650	0.945
BCGP-Doc2vec	0.413	0.895	0.381	0.780	0.545	0.932	0.652	0.944
BCGP-HyenaDNA	0.427	0.894	0.393	0.789	0.542	0.940	0.663	0.948
BCGP-BERT	**0.439**	**0.904**	**0.435**	**0.816**	**0.572**	**0.948**	**0.712**	**0.957**

The best results of four evaluation metrics (F1, AUC, AP, and NDCG) are highlighted in bold.

### 3.3 Examination of fine-tuning

To evaluate the performance of BCGP integrated with different fine-tuning methods, we conduct association prediction experiments using six different GNNs. Specifically, we compare GAT (Veličković et al., 2017), GATv2 (Brody et al., 2021), FiLM (Brockschmidt, 2020), GraphSAGE (Hamilton et al., 2017), SGC (Wu et al., 2019), and GCN (Kipf and Welling, 2016) using the BCGP-BERT pre-training framework. All methods share the same embedding dimension. As detailed in Table 2, for the lncRNA-miRNA association prediction, GCN outperforms all other fine-tuning methods, achieving an F1 score of 0.439, AUC of 0.904, AP of 0.435, and NDCG of 0.816. For the circRNA-miRNA association prediction, GCN also demonstrates a robust performance with an F1 score of 0.572, AUC of 0.948, AP of 0.712, and NDCG of 0.957, while SGC closely follows with competitive performance. These results demonstrate that GCN is the most effective fine-tuning method on BCGP-BERT for predicting lncRNA-miRNA and circRNA-miRNA interactions. Further fine-tuning results on the LMA2 and CMA2 datasets are provided in Supplementary Appendix S3.

**TABLE 2:** Overall performance comparison of different fine-tuning GNN methods on lncRNA-miRNA and circRNA-miRNA association prediction tasks using the LMA1 and CMA1 datasets.

Methods	lncRNA-miRNA				circRNA-miRNA			
Fine-tune	F1	AUC	AP	NDCG	F1	AUC	AP	NDCG
GAT	0.310	0.768	0.211	0.697	0.462	0.919	0.527	0.910
GATv2	0.329	0.723	0.187	0.685	0.415	0.884	0.430	0.887
FiLM	0.443	0.894	0.367	0.772	0.473	0.931	0.612	0.939
GraphSAGE	0.302	0.756	0.202	0.691	0.483	0.935	0.620	0.941
SGC	0.399	0.895	0.421	0.818	0.571	0.947	0.698	0.954
GCN	**0.439**	**0.904**	**0.435**	**0.816**	**0.572**	**0.948**	**0.712**	**0.957**

The pre-training method used is BCGP-BERT. The best results of four evaluation metrics (F1, AUC, AP and NDCG) are highlighted in bold.

### 3.4 Method comparison

Next, we compare our BCGP to the state-of-the-art SPGNN (Wang Z. et al., 2023), which utilizes the k -mer technique, Doc2Vec model and fine-tuning with GNN for RNA association prediction. Additionally, we include GCNFormer (Yao et al., 2024), which leverages graph convolutional networks and transformers for predicting lncRNA-disease associations, as a baseline. The experimental results in Table 3 illustrate that our BCGP consistently outperforms SPGNN across all metrics for both lncRNA-miRNA and circRNA-miRNA association predictions. For lncRNA-miRNA associations, our BCGP achieves an F1 score of 0.439, AUC of 0.903, AP of 0.435, and NDCG of 0.812, outperforming SPGNN’s scores of 0.430, 0.894, 0.419, and 0.828, respectively. Similarly, for circRNA-miRNA, BCGP obtains an F1 score of 0.572, AUC of 0.948, AP of 0.712, and NDCG of 0.957, significantly outperforming SPGNN. Additionally, we evaluate the effect of the NCN technique on the performance of BCGP. Compared to the BCGP without NCN, the integration of NCN shows improvements in both lncRNA-miRNA and circRNA-miRNA association predictions. These results demonstrate that BERT has a stronger capability to capture contextual information than the classic Doc2vec method, which allows our method to effectively capture complex and intricate relationships between RNA sequences, leading to more accurate predictions. Furthermore, integrating the NCN technique enables our method to learn more refined node representations, which ultimately enhances the prediction accuracy in complex RNA interactions.

**TABLE 3:** Overall performance comparison of SPGNN and our BCGP method on lncRNA-miRNA-circRNA association prediction task.

Methods	lncRNA-miRNA				circRNA-miRNA			
	F1	AUC	AP	NDCG	F1	AUC	AP	NDCG
SPGNN (Wang et al., 2023b)	0.430	0.894	0.419	**0.828**	0.403	0.840	0.510	0.911
GCNFormer (Yao et al., 2024)	0.305	0.677	0.226	0.686	0.350	0.730	0.453	0.815
BCGP (w/o NCN)	$0.432^{†}$	$0.901^{†}$	$0.432^{†}$	0.808	$0.492^{†}$	$0.919^{†}$	$0.577^{†}$	$0.929^{†}$
BCGP (w/NCN)	**0.439** †	**0.903** †	**0.435** †	0.812	**0.572** †	**0.948** †	**0.712** †	**0.957** †

The best results of four evaluation metrics (F1, AUC, AP, and NDCG) are highlighted in bold. In each dataset, significant improvements over the base model are marked with † (paired t-test, p < 0.05$).

### 3.5 Analysis of hyperparameters

In this section, we investigate the impacts of two important parameters of the BCGP, including the k -value in k -mers and the RNA embedding vector size in the pre-training stage.

#### 3.5.1 Examination of k-value

**FIGURE 2:**
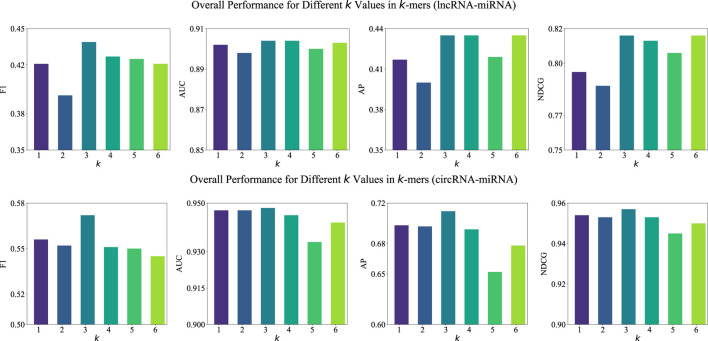
Overall performance (F1, AUC, AP, and NDCG) of BCGP-BERT-GCN across varying k values in k-mers for lncRNA-miRNA (first row) and circRNA-miRNA (second row) association predictions.

In our BCGP method, we employ the k -mers tokenization to segment RNA sequences into equal-length segments, where k denotes the segment length. To determine the impact of k value, we investigate the performance of BCGP across different k values, limiting k to a maximum of 6 due to computational constraints. As illustrated in Figure 2, the experimental results indicate that k=3 provides the optimal performance for both lncRNA-miRNA and circRNA-miRNA association predictions. For lncRNA-miRNA associations, the k=3 setting consistently achieves the highest F1, AP, and NDCG scores. Similarly, for circRNA-miRNA associations, k=3 obtains the highest scores for F1 and NDCG. These results suggest that the choice of k=3 can help BCGP capture sufficient sequence context and identify the most informative patterns for RNA association predictions.

#### 3.5.2 Examination of embedding size in the pre-training stage

**FIGURE 3:**
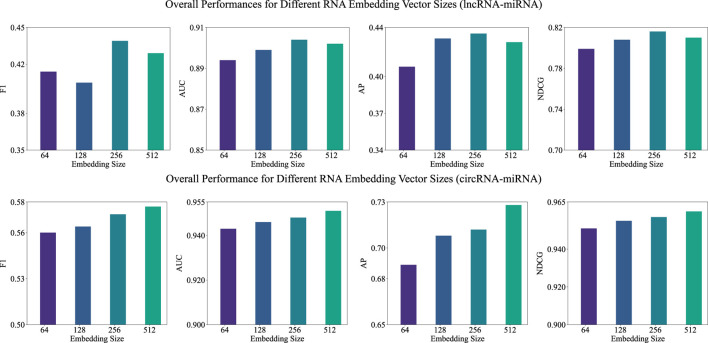
Overall performance (F1, AUC, AP, and NDCG) of BCGP-BERT-GCN across varying embedding sizes for lncRNA-miRNA (first row) and circRNA-miRNA (second row) association predictions.

After analyzing the impact of k value, we examine the effect of RNA embedding vector size within the BERT model during the pre-training stage. Due to the memory constraint, we vary the embedding size from 64 to 512 and fixed the k -value in k -mers at 3. As depicted in Figure 3, the embedding size significantly impacts the performance of both lncRNA-miRNA and circRNA-miRNA association predictions. For lncRNA-miRNA, the optimal performance is achieved with an embedding size of 256. In contrast, for circRNA-miRNA, we can observe a consistent improvement across all metrics with increasing embedding sizes. The findings suggest that while larger embedding sizes tend to enhance the ability of the model to capture complex interactions, the optimal embedding size may vary between datasets.

### 3.6 Case study

Prediction of new circRNA-miRNA and lncRNA-miRNA interactions can reveal new biomarkers, identify therapeutic targets, and enhance understanding of the regulatory mechanisms of biological networks. To validate the practicality of our method, we select two miRNAs, namely hsa-miR-143 and hsa-miR-6808-5p, to verify the prediction results of miRNA-lncRNA and miRNA-circRNA associations generated by BCGP. External literature is employed to validate the predictions for miRNA-lncRNA associations, while associations recorded in CircBank (Liu et al., 2019) are used for verifying miRNA-circRNA associations. For the target miRNAs, 8 out of the top-10 predicted miRNA-lncRNA associations are confirmed in Pubmed, and 9 out of the top 10 miRNA-circRNA associations are validated in Circbank.

Using the BCGP model, we predict lncRNAs linked to hsa-miR-143. As illustrated in Table 4, the top-10 predicted lncRNAs are MALAT1, MEG3, NEAT1, UCA1, DANCR, HOTAIR, TUG1, GAS5, KCNQ1OT1, and MIAT, with 8 of these associations being validated in external literature. For instance, MALAT1 the top-ranked lncRNA, was shown in a study by Chen et al. (2017) to regulate ZEB1 expression by sponging miR-143-3p and promoting the progression of Hepatocellular Carcinoma. Additionally, Dong et al. (2020) has demonstrated that MEG3 overexpression inhibited LPS-induced injury in PDLCs by deactivating the AKT/IKK pathway by sponging miR-143-3p.

**TABLE 4:** Top-10 Predicted lncRNAs Linked to hsa-miR-143.

Rank	lncRNA	PMID
1	MALAT1	28,543,721
2	MEG3	32,520,926
3	NEAT1	33,744,906
4	UCA1	32,130,788
5	DANCR	Not found
6	HOTAIR	29,336,659
7	TUG1	31,264,280
8	GAS5	36,769,379
9	KCNQ1OT1	30,691,798
10	MIAT	Not found

Similarly, we predict circRNAs that are potentially associated with hsa-miR-6808-5p validated by CircBank. The results presented in Table 5 reveal that the top-10 ranked circRNAs as hsa_circ_0082878, hsa_circ_0020316, hsa_circ_0049111, hsa_circ_0057955, hsa_circ_0037997, hsa_circ_0000726, hsa_circ_0049109, hsa_circ_0049112, hsa_circ_0016773, and hsa_circ_0085900. Upon searching CircBank for hsa-miR-6808-5p, we find 9 out of the top 10 predicted results in the CircBank dataset. The results of the case study indicate that BCGP possesses commendable practicality.

**TABLE 5:** Top-10 Predicted lncRNAs Linked to hsa-miR-6808-5p.

Rank	lncRNA	Evidence
1	hsa_circ_0082878	Found
2	hsa_circ_0020316	Found
3	hsa_circ_0049111	Found
4	hsa_circ_0057955	Found
5	hsa_circ_0037997	Found
6	hsa_circ_0000726	Found
7	hsa_circ_0049109	Found
8	hsa_circ_0049112	Found
9	hsa_circ_0016773	Found
10	hsa_circ_0085900	Not Found

## 4 Conclusion

In this article, we propose a novel method named BCGP, to leverage RNA sequence information and heterogeneous relationships to enhance the prediction of lncRNA-miRNA and circRNA-miRNA associations. To comprehensively capture contextual and structural information, BCGP integrates BERT in the pre-training stage to consider the global context of the entire sequence. To further enhance the performance of association prediction, BCGP leverages the Neural Common Neighbour technique in the fine-tuning stage to learn more informative and flexible representations. Extensive experiments on two real-world benchmark datasets demonstrate the effectiveness of our BCGP, showing that it significantly improves prediction accuracy by capturing complex interactions in both lncRNA-miRNA and circRNA-miRNA association prediction tasks compared with competitive baselines.

## Data Availability

The original contributions presented in the study are included in the article/Supplementary Material, further inquiries can be directed to the corresponding author.
