# Supplementary material for: Integrating BERT pre-training with graph common neighbours for predicting ceRNA interactions
Source: Front Genet. 2025 Sep 3;16:1606016. doi: 10.3389/fgene.2025.1606016 (PMC12441610; doi:10.3389/fgene.2025.1606016)
Supplement: Supplementary file 1 [file Presentation1.pdf]

Supplementary Material for Integrating BERT Pre-training with Graph Common Neighbours for Predicting ceRNA Interactions

Zhengxing Xie [1†], **Tianping Yin** [2†], **Ge Jing** [2], **Shiyang Liang** [3], **Junhua Liu** [4] **and Lianghua Tang** [2*]

[1] *Department of Joint Surgery, Guizhou University of Traditional Chinese Medicine, Guizhou 550002, China*
[2] *Department of Joint Surgery, The Second Affiliated Hospital of Guizhou University of Traditional Chinese Medicine, Guizhou 550001, China*
[3] *Department of Internal Medicine, The No. 944 Hospital of Logistic Support Force of PLA, Jiuquan, Gansu, China*
[4] *The University of Melbourne, Melbourne, Australia*
[†] *These authors have contributed equally to this work and share first authorship.*

Correspondence*:
Lianghua Tang (TLH13985059303@outlook.com), The Second Affiliated Hospital of Guizhou University of Traditional Chinese Medicine, No. 32 Feishan Street, Yunyan District, Guizhou 550001, China.

1 EVALUATION METRICS

Following the evaluation metrics used in previous works (Wang et al., 2023; Guo et al., 2024), we use the F1 score, the Area Under the Receiver Operating Characteristic Curve (AUC), Average Precision (AP) and Normalized Discounted Cumulative Gain (NDCG) (Järvelin and Kekäläinen, 2002) as the metrics to evaluate the performance of lncRNA-miRNA and circRNA-miRNA association prediction results.

The F1 score is a widely used metric in binary classification, considering precision and recall to comprehensively evaluate a model's accuracy. The combination of these two metrics into a single value allows the F1 score to offer a balanced assessment of model performance. Specifically, the definitions of precision, recall, and F1 score are given as follows:

$$\text{Precision} = \frac{\text{TP}}{\text{TP} + \text{FP}} \tag{1}$$

$$\text{Recall} = \frac{\text{TP}}{\text{TP} + \text{FN}} \tag{2}$$

$$\text{F1 Score} = \frac{2 \times \text{Precision} \times \text{Recall}}{\text{Precision} + \text{Recall}}\,, \tag{3}$$

where TP, FP, and FN are denoted as true positive, false positive, and false negative.

The AUC is a metric used to evaluate the accuracy of a binary classification model. It measures the performance by comparing the true positive rate (TPR) and false positive rate (FPR).

Specifically, the TPR and FPR are defined as follows:

$$\text{TPR} = \frac{\text{TP}}{\text{TP} + \text{FN}} \tag{4}$$

$$\text{FPR} = \frac{\text{FP}}{\text{FP} + \text{TN}} . \tag{5}$$

This comparison is visually represented through the receiver operating characteristic (ROC) curve, which plots the TPR against the FPR at various classification thresholds. The AUC represents the area under the ROC curve, comprehensively evaluating the performance across all thresholds.

The average precision (AP) serves as another performance metric for evaluating the accuracy of the classifier in our task. It is derived by computing the weighted average of precision across various thresholds on the precision-recall curve, where the weight is determined by the increase in recall from the previous threshold. Specifically, the AP is defined as follows:

$$\text{AP} = \sum_{i=1}^{n} (R(i) - R(i-1)) \cdot P(i) , \tag{6}$$

where $P(i)$ represents the precision at the i-th threshold, $R(i)$ represents the recall at the i-th threshold.

The NDCG evaluates ranked lists by considering the relevance of items and their positions in the ranking. It calculates the Discounted Cumulative Gain (DCG) by summing the discounted relevance scores of items, emphasizing the importance of higher-ranked positions. NDCG normalizes this score by the ideal DCG (IDCG), the maximum DCG achievable with a perfect ranking, which measures how close the actual ranking is to the ideal. This normalization makes NDCG a comprehensive metric that accounts for the relevance and order of items, offering a more detailed assessment ranking quality than simpler metrics like precision or recall. Specifically, the DCG, IDCG and NDCG are defined as follows:

$$\text{DCG}_p = \sum_{i=1}^{p} \frac{2^{\text{rel}_i} - 1}{\log(i+1)} \tag{7}$$

$$\text{IDCG}_p = \sum_{i=1}^{p} \frac{2^{\text{rel}_i} - 1}{\log(i+1)} \tag{8}$$

$$\text{NDCG}_p = \frac{\text{DCG}_p}{\text{IDCG}_p} , \tag{9}$$

where the p refers to the position up to which the ranking is evaluated, and ref refers to the relevance score based on its rank i. The IDCG is computed similarly to DCG but under an ideal ranking order, where items are sorted by their relevance in descending order.

In line with the previous work Wang et al. (2023); Guo et al. (2024), we evaluate the performance of our BCGP using K-fold cross-validation, where we set K to 5. Specifically, we randomly divide the lncRNA-miRNA-circRNA associations dataset into five equal-sized subsets, using one as the testing set and the rest as the training set. To ensure the reliability of

the training and validation sets, we randomly select 10 unobserved edges from the lncRNA-miRNA-circRNA association graph as negative samples so that the positive-negative ratio is 1:10. This sampling process is performed separately for lncRNA-miRNA and circRNA-miRNA interactions using PyTorch Geometric's negative sampling implementation to maintain sampling consistency across different RNA interaction types. This process is repeated five times to ensure the consistency and stability of the evaluation results. Finally, we average the cross-validation results for a single comprehensive estimation, which measures the overall performance of our method and all other baselines.

2 EXPERIMENTAL SETTING

To optimize our method, we employ the Adam optimizer (Kingma and Ba, 2015) with a learning rate of 0.01. To guarantee the reproducibility of our results, we have fixed the seed value at 42, ensuring consistency across the various random operations within our method. We evaluated BCGP on NVIDIA RTX 3080 (10GB), where complete training finished within 2 hours using batch size 768. The method operates within memory constraints while maintaining practical training times on consumer hardware. We utilize the GNN module implementations provided by PyTorch Geometric (Fey and Lenssen, 2019), and implement BCGP using PyTorch (Paszke et al., 2017).

Building upon the previous works (Dutta et al., 2018; Zhang et al., 2021; Wang et al., 2023), our approach to the pre-training stage for embedding RNA sequences employs a consistent k-mer method with a fixed k-value of 3. our implementation of the pre-training stage for RNA sequence embedding adopts a consistent k-valued of 3 in the k-mer method. To justify our choice of k value, we vary it from 1 to 6, where our ablation study reveals that 3 is the best choice.Hence, lncRNA, circRNA, and miRNA sequences are tokenized into 3-mers. We then focus on exploring the BERT model and compare it with other pre-training methods to demonstrate the necessity and effectiveness of pre-training within our proposed method BCGP. The following parameters of the BERT model are utilized as the default hyperparameters in our experiments to obtain optimal performance. The RNA embedding dimension is set to 256, the number of layers is set to 4, and the number of attention heads is set to 4. The BERT model is trained for 100 epochs, which is the number of iterations over the entire corpus. After obtaining the pre-trained embedding for each node, we normalize them to enhance the effectiveness of the link prediction task in the subsequent fine-tuning stage.

In the fine-tuning stage, we employ the GCN model as the default model architecture, which consists of GCN layers with ReLU activation in between. As a default setting in our experiments, we set the node features to 256 and dimension to 128 for the rest of the GNN models, including GraphSAGE, GAT, GATv2, FiLM, and SGC. For the sake of brevity, we use BCGP to denote BCGP-BERT-GCN, which is the best-performing variant.

3 EXPERIMENTS ON LMA2 AND CMA2

To assess the generalizability of our proposed method, we conducted additional experiments using datasets LMA2 and CMA2. Specifically, the lncRNA-miRNA associations in LMA2 were sourced from lncRNASNP V3.0 (Yang et al., 2023), comprising a total of 8,502 lncRNA-miRNA associations, including 467 lncRNAs and 254 miRNAs. As for the circRNA-miRNA associations in CMA2, they were derived from the dataset 1 of the KGANCDA (Lan et al.,

**Methods**	**lncRNA-miRNA**				**circRNA-miRNA**			
**Pre-train**	**F1**	**AUC**	**AP**	**NDCG**	**F1**	**AUC**	**AP**	**NDCG**
BCGP-Random	0.431	0.902	0.513	0.906	0.309	0.735	0.133	0.617
BCGP-Text2vec	0.584	0.949	0.652	0.940	0.319	0.772	0.172	0.643
BCGP-Doc2vec	0.478	0.918	0.588	0.926	0.327	0.773	0.175	0.633
BCGP	**0.661**	**0.968**	**0.729**	**0.955**	**0.329**	**0.779**	**0.179**	**0.649**

Table 1. Overall performance comparison of different pre-training methods fine-tuned with GCN on lncRNA-miRNA and circRNA-miRNA association prediction tasks using the LMA2 and CMA2 datasets. The best results of four evaluation metrics (F1, AUC, AP, and NDCG) are highlighted in bold.

**Methods**	**lncRNA-miRNA**				**circRNA-miRNA**			
**Fine-tune**	**F1**	**AUC**	**AP**	**NDCG**	**F1**	**AUC**	**AP**	**NDCG**
GAT	0.391	0.870	0.307	0.834	0.315	0.774	0.218	0.678
GATv2	0.435	0.877	0.338	0.846	0.326	**0.785**	0.225	0.684
FiLM	0.556	0.942	0.509	0.894	0.284	0.612	0.104	0.573
GraphSAGE	0.554	0.944	0.543	0.909	**0.392**	0.746	0.**287**	**0.725**
SGC	0.658	0.967	**0.730**	**0.955**	0.335	0.776	0.175	0.646
GCN	**0.661**	**0.968**	0.729	**0.955**	0.329	0.779	0.179	0.649

Table 2. Overall performance comparison of different fine-tuning GNN methods on lncRNA-miRNA and circRNA-miRNA association prediction tasks using the LMA2 and CMA2 datasets. The pre-training method used is BCGP-BERT. The best results of four evaluation metrics (F1, AUC, AP and NDCG) are highlighted in bold.

2022), with a total of 702 circRNA-miRNA associations, encompassing 471 circRNAs and 439 miRNAs. The sequences of lncRNAs were sourced from LNCipedia (Volders et al., 2019) and NONCODE (Zhao et al., 2021), the sequences of circRNAs were obtained from CircBase (Glažar et al., 2014), and the sequences of miRNAs were acquired from miRBase (Griffiths-Jones et al., 2007). The experimental results are summarized in Table 1 and Table 2.

REFERENCES

Dutta, A., Dubey, T., Singh, K. K., and Anand, A. (2018). Splicevec: distributed feature representations for splice junction prediction. *Computational biology and chemistry* 74, 434–441

Fey, M. and Lenssen, J. E. (2019). Fast graph representation learning with pytorch geometric. In *ICLR 2019 (RLGM Workshop)*

Glažar, P., Papavasileiou, P., and Rajewsky, N. (2014). circbase: a database for circular rnas. *Rna* 20, 1666–1670

Griffiths-Jones, S., Saini, H. K., Van Dongen, S., and Enright, A. J. (2007). mirbase: tools for microrna genomics. *Nucleic acids research* 36, D154–D158

Guo, L.-X., Wang, L., You, Z.-H., Yu, C.-Q., Hu, M.-L., Zhao, B.-W., et al. (2024). Likelihood-based feature representation learning combined with neighborhood information for predicting circrna–mirna associations. *Briefings in Bioinformatics* 25, bbae020

Järvelin, K. and Kekäläinen, J. (2002). Cumulated gain-based evaluation of ir techniques. *ACM Transactions on Information Systems (TOIS)* 20, 422–446

Kingma, D. P. and Ba, J. (2015). Adam: A method for stochastic optimization. In *International Conference on Learning Representations*

Lan, W., Dong, Y., Chen, Q., Zheng, R., Liu, J., Pan, Y., et al. (2022). Kgancda: predicting circrna-disease associations based on knowledge graph attention network. *Briefings in Bioinformatics* 23, bbab494

Paszke, A., Gross, S., Chintala, S., Chanan, G., Yang, E., DeVito, Z., et al. (2017). Automatic differentiation in pytorch

Volders, P.-J., Anckaert, J., Verheggen, K., Nuytens, J., Martens, L., Mestdagh, P., et al. (2019). Lncipedia 5: towards a reference set of human long non-coding rnas. *Nucleic acids research* 47, D135–D139

Wang, Z., Liang, S., Liu, S., Meng, Z., Wang, J., and Liang, S. (2023). Sequence pre-training-based graph neural network for predicting lncrna-mirna associations. *Briefings in Bioinformatics* 24, bbad317

Yang, Y., Wang, D., Miao, Y.-R., Wu, X., Luo, H., Cao, W., et al. (2023). lncrnasnp v3: an updated database for functional variants in long non-coding rnas. *Nucleic Acids Research* 51, D192–D198

Zhang, Y., Liu, Y., Xu, J., Wang, X., Peng, X., Song, J., et al. (2021). Leveraging the attention mechanism to improve the identification of dna n6-methyladenine sites. *Briefings in Bioinformatics* 22, bbab351

Zhao, L., Wang, J., Li, Y., Song, T., Wu, Y., Fang, S., et al. (2021). Noncodev6: an updated database dedicated to long non-coding rna annotation in both animals and plants. *Nucleic acids research* 49, D165–D171
